# Combination forecasting of COVID-19 in Bangladesh: guiding public health policy through integrated time series, ML, and DL models

**DOI:** 10.1186/s12879-025-12305-3

**Published:** 2025-12-12

**Authors:** Sharmin Akther, Amartay Kumar Dhar, Jakia Sultana Pingky

**Affiliations:** Department of Statistics and Data Science, Jahangirnagar University, Dhaka, 1342 Bangladesh

**Keywords:** COVID-19 forecasting, Hybrid modeling, Machine learning, Deep learning, Time series analysis, Public health surveillance

## Abstract

**Background:**

Accurate prediction of trends in infectious diseases is crucial for early public health actions, which are difficult to achieve in low-resource countries such as Bangladesh, where the healthcare system is significantly challenged. Although many researchers worldwide have utilized machine learning (ML) and deep learning (DL) models for making COVID-19 predictions, limited research has focused on context-specific ensemble forecasting approaches for Bangladesh, creating a gap in local epidemic modeling. This study aims to design a Weighted Ensemble forecasting model that combines the best time series models (SARIMA, ETS), ML (XGBoost), and DL (LSTM, RNN, GRU) models to improve the accuracy of daily new COVID-19 cases in Bangladesh.

**Methods:**

We developed a context-specific Weighted Ensemble model integrating Seasonal Autoregressive Integrated Moving Average, XGBoost, and Recurrent Neural Network, with weights (0.1531, 0.4319, 0.4150). Log transformation handled zeros, with scaling for neural networks. Forecasts extended to May 2027.

**Result:**

Out-of-sample results (SARIMA: RMSE = 14.56, MAE = 9.02, MAPE = 216.87%; XGBoost: RMSE = 5.16, MAE = 3.45, MAPE = 134.52%; Recurrent Neural Network: RMSE = 5.37, MAE = 2.71, MAPE = 41.11%; Weighted Ensemble: RMSE = 5.87, MAE = 3.49, MAPE = 111.02%) show the Weighted Ensemble outperforms individual models, including the best time series, machine learning, and deep learning models, despite misspecification in traditional models.

**Conclusions:**

The Combination model based on Seasonal Autoregressive Integrated Moving Average, XGBoost, and Recurrent Neural Network can improve prediction performance for Bangladesh’s complex COVID-19 data better than the XGBoost model does. The power of ensemble modeling in capturing linear seasonality and the nonlinear dynamics it is evident from these results. This novel integration can inform public health policy, allowing health authorities to fine-tune interventions and make more efficient use of resources while avoiding unnecessary lockdowns as COVID-19 becomes an endemic disease.

## Introduction

The COVID-19 pandemic, caused by the SARS-CoV-2 virus, has been one of the most significant global health crises of the 21st century [1]. Since it was first reported in late 2019, COVID-19 has rapidly spread worldwide, affecting millions of people and disrupting economies, healthcare systems, and daily life [2]. As of mid-2025, the global toll of the pandemic stands at over 700 million confirmed cases, with more than 7 million deaths [3]. While the development and distribution of vaccines have slowed the spread of the virus, the pandemic is far from over [4]. Variants of concern continue to emerge, and new surges in cases are reported periodically, highlighting the ongoing need for robust forecasting and prediction models to anticipate future trends and guide public health policies [5]. In the 28 days from 2 to 29 June, 2025, 74 countries across five WHO regions reported 307,833 new COVID-19 cases, a 2% increase compared with the previous period. Additionally, 37 countries reported 1,010 new deaths during the same period [6]. Despite widespread vaccination campaigns, some countries, including the United States, India, and Brazil, continue to report high infection rates [3]. Countries in Africa and Southeast Asia have also faced challenges due to limited healthcare infrastructure and vaccination access, demonstrating global inequalities in pandemic management [7].

In Bangladesh, the impact of COVID-19 has been equally devastating. The country reported its first confirmed case in March 2020, and since then, it has experienced rapid and widespread infection [8]. As of 2025, Bangladesh has reported over 2 million confirmed cases and more than 29,000 deaths, although the true numbers may be higher due to limited testing and reporting during the peak of the pandemic [9]. The healthcare system, which is already burdened by preexisting challenges, was overwhelmed during the worst phases of the crisis [8]. Despite government measures, including lockdowns and vaccination campaigns, the continuous threat of new outbreaks has raised concerns about the country’s ability to manage future waves [10]. The economic consequences have been profound, with many sectors experiencing dramatic declines and millions facing unemployment and poverty [11]. In 2025 alone, Bangladesh reported 665 new cases and 27 deaths from January 1 to July 16 [9].

In response to these challenges, various machine learning (ML) and deep learning (DL) models have been employed to predict the trajectory of COVID-19 cases and deaths, each contributing valuable insights to pandemic forecasting [12, 13]. Machine learning techniques such as vector machines (SVMs), random forests, and XGBoost have been widely used for their ability to model complex, nonlinear relationships and process large datasets. For example, multiple studies have demonstrated that XGBoost can effectively predict the number of COVID-19 cases by incorporating diverse variables, such as government interventions, mobility data, and population density [12, 14]. Deep learning models, particularly long short-term memory (LSTM) networks, have gained traction because of their effectiveness in handling time series data, capturing complex temporal dependencies in COVID-19 infection rates. LSTM-based models have been shown to outperform traditional time series models such as ARIMA in predicting future trends [15]. Additionally, gated recurrent units (GRUs), another type of recurrent neural network, have also been applied to predict COVID-19 cases, showing improved accuracy over simpler ML models [16]. Hybrid approaches that combine ML and DL models have been further explored to improve forecast accuracy by leveraging the strengths of both techniques [17]. These models have demonstrated a significant improvement over traditional models, offering more robust and adaptable predictions. Although challenges remain in terms of data quality and prediction uncertainty, recent advances such as Shapley value–based methods have improved the interpretability of machine learning models by quantifying the relative importance of input features [18].

Despite the growing interest in machine learning and deep learning applications for COVID-19 prediction, there remains a significant gap in research focused specifically on Bangladesh [14]. While global studies have explored the use of ML and DL models for pandemic forecasting, most of them have concentrated on countries with more robust healthcare infrastructures and data availability [19]. In contrast, Bangladesh’s unique socioeconomic, healthcare, and data challenges require specialized models [20]. Furthermore, much of the existing research has focused on isolated models rather than the integration of multiple approaches. To this end, we propose a weighted ensemble framework that combines various statistical/ML models for COVID-19 case forecasts specific to Bangladesh and yields better, smoother, and context-sensitive predictions compared to existing approaches.

This study aims to integrate several best forecasting methods, i.e., time series analysis, machine learning (ML), and deep learning (DL) models for the prediction of future trends in COVID-19 cases in Bangladesh. In this work, we propose to utilize the merits of these models in a weighted ensemble approach for producing accurate and trustworthy daily new cases forecasts. The forecasts are relevant from June 2025 to May 2027, which offer a detailed visibility on possible future surges. These forward-looking results are intended to guide public health officials in strategic planning, enabling the timely allocation of medical resources, strengthening preparedness measures, and minimizing the need for disruptive interventions such as nationwide lockdowns.

## Methods and materials

### Data source

This study uses historical data of daily new COVID-19 cases in Bangladesh from March 2020 to May 2025. The dataset includes important features such as new cases, dates, and seasonality factors. This study utilized data obtained from the “Our World in Data” website (https://ourworldindata.org/). The analysis was conducted via Python version 3.12.10.

### Data preprocessing

This study was based on the daily new COVID-19 cases feature only, making up a univariate time series (1,905 daily observations from March 2020 to May 2025) from Our World in Data. The data set had 2.94 percent of zero values, which artificially inflated MAPE. To curb this, a log1p transformation (log (1 + x)), where x is the daily new case count was used, and Python 3.12.10 was used to stabilize the variance and allow the use of zeros to reduce skew due to high variability (e.g., peaks of 16,000 cases). The log-transformed series was then scaled to [0,1] with MinMaxScaler to stabilize neural network-based models (e.g., LSTM, GRU, RNN) to improve the overall model performance.

### SARIMA model

The Seasonal Autoregressive Integrated Moving Average (SARIMA) model is an extension of the ARIMA model designed to handle time series data with seasonal effects [21]. It is represented as ARIMA (p, d, q) (P, D, Q)s, where p, d, and q are the nonseasonal components (autoregressive, differencing, and moving average orders), respectively, and P, D, Q, and s are the seasonal components (seasonal autoregressive, seasonal differencing, seasonal moving average, and the length of the seasonal period, respectively) [22]. The SARIMA model captures both nonseasonal and seasonal patterns by combining these components into a single equation, allowing it to forecast data that exhibit periodic fluctuations, such as monthly, quarterly, or yearly trends [23].

### ETS model

The Error, Trend, Seasonality (ETS) model, which accounts for seasonal and trend elements, is a method used to forecast univariate time series data [24]. This model is highly versatile and capable of generating seasonal components for a broad range of properties and trends [25]. The model is defined by three main components: error, trend, and seasonality. Each component can have four possible settings: *A* for additive, *M* for multiplicative, *N* for none, and *Z* for auto. ETS (*A, M, N*) represents an additive error, a multiplicative trend, and no seasonal component [26]. We first tested an ETS (A, A, A) with 7 days on log-transformed data. Hashed occurred corresponding to seasonality (multiplicative seasonality, e.g., 16,000 case spikes in 2021–2022), so an ETS (A, A, M) adjustment was made. The Ljung-Box test detected residual autocorrelation (*p* = 0.0000, lag 1), which indicated non-linearity problems and could be associated with the 2.94% zero values; however, it was resolved with model integration.

### XGBoost model

XGBoost is a highly effective machine learning algorithm widely used for time series forecasting because of its ability to capture complex, seasonal, and nonlinear trends. It constructs an ensemble of decision trees for regression to predict outcomes, reducing the error between the true and predicted values while controlling the complexity of the trees. XGBoost is adept at modeling complex relationships, learning nonlinear interactions, and addressing outliers. Additionally, it enables advanced feature engineering, allowing for the integration of external factors to enhance forecasting precision [27, 28].

### LSTM model

Long short-term memory (LSTM) networks are a type of recurrent neural network (RNN) designed to better capture long-term dependencies in sequential data [29]. Unlike traditional RNNs, LSTMs address the vanishing gradient problem by using special memory cells, which allow the network to remember information for long periods. Each LSTM unit contains three gates, input, forget, and output, that regulate the flow of information. The input gate decides which values are updated, the forget gate determines which information to discard, and the output gate decides which information will be passed to the next time step. These gates enable LSTMs to effectively learn from sequences, making them highly suitable for tasks such as time series forecasting, speech recognition, and natural language processing, where long-term dependencies are crucial [30].

### RNN model

Recurrent neural networks (RNNs) are designed for sequential data, where the output at each time step is influenced by previous inputs [31, 32]. RNNs work by maintaining a hidden state that captures information about the sequence processed thus far, making them ideal for tasks where time-dependent relationships are important. However, basic RNNs struggle with learning long-term dependencies due to the vanishing gradient problem, where gradients used for training diminish as they are propagated back through many time steps, limiting their ability to learn from long sequences. Despite this limitation, RNNs are still useful for many applications, such as text generation and speech recognition, where data have a sequential structure but dependencies are not necessarily long-term [32].

### Gru model

Compared with LSTMs, gated recurrent units (GRUs) are variants of RNNs that aim to overcome the limitations of traditional RNNs by using fewer gates and simplifying the structure. GRUs combine the input and forget gates of an LSTM into a single “update gate,” which determines how much of the past information to keep and how much to discard. They also use a reset gate to decide how much of the previous hidden state to forget. GRUs are less computationally expensive than LSTMs while still being able to capture dependencies in sequential data effectively. This makes them a popular choice in applications such as time series prediction, video analysis, and speech recognition, where they can outperform traditional RNNs while requiring less computational power [33–35].

### Evaluation metrics

**Fig. 1:**
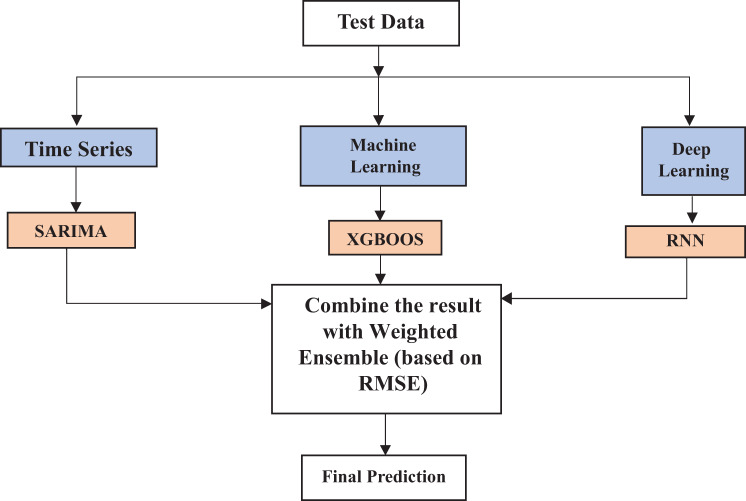
Architectural design of the proposed combined model

The performance of the combination models was evaluated by the root mean square error (RMSE), mean absolute error (MAE), mean absolute percentage error (MAPE), first-lag autocorrelation of residuals (ACF1), and mean absolute scaled error (MASE). Each measure was calculated via the following formulas:

1 $$RMSE = \sqrt {{1 \over n}\mathop \sum \limits_{i = 1}^n {{\left( {{y_i} - {{\hat y}_i}} \right)}^2}} $$

2 $$MAE = {1 \over n}\mathop \sum \limits_{i = 1}^n \left| {{y_i} - {{\hat y}_i}} \right|$$

3 $$MAPE = {1 \over n}\mathop \sum \limits_{i = 1}^n {{\left| {{y_i} - {{\hat y}_i}} \right|} \over {{y_i}}} \times 100\% $$

4 $$\,MASE = \frac{1}{n}\mathop \sum \limits_{i = 1}^n \left( {\frac{{\left| {{y_i} - {{\hat y}_i}} \right|}}{{\frac{1}{{n - m}}\,\mathop \sum \nolimits_{j = m + 1}^n \left| {{y_j} - {y_{j - m}}} \right|\,}}} \right)$$

where n indicates the number of observations, ${y_i}$ denotes the original values, ${\hat y_i}$ represents the predicted values, and m is the seasonality value. MAE and RMSE are scale-dependent metrics that are based on absolute errors and squared errors, respectively. The MAPE is a unit-free error measure based on percentage errors, and the MASE is a scale-free error metric. ACF1 is also defined as

5 $$ACF1 = Corr\left( {{e_t},{e_{t - 1}}} \right),$$

where ${e_t} = {y_t} - {\hat y_t}$ is the residual at time *t*. An ACF1 close to zero suggests that the residuals are random and that the model has captured the underlying time dependence well. The flowchart of this study is shown in Fig. 1. Figure 1 represents the integrated model, a combination of the best time series model (SARIMA), machine learning model (XGBoost), and deep learning model (RNN) chosen according to the test data results, by using a weighted assembly. Calculation of weights is done by dividing the error measure of each model by its summation in a normal way to one.

Various statistical, machine learning, and deep learning models were developed in this research to predict the number of COVID-19 cases per day. The time series was initially log1p scaled to stabilize variance and reduce skew, and further scaled to the [0,1] range by MinMaxScaler to enhance convergence and better performance of the neural network models. In the case of XGBoost, the hyperparameters such as the number of estimators (n_estimators), the maximum depth of the tree (max_depth), and the learning rate (learning rate) were optimized through a grid search, and subsampling (subsample) and column sampling (colsample_bytree) were kept fixed to alleviate overfitting. The final model was chosen as the hyperparameter combination that produces the lowest value of root mean squared error (RMSE) on the validation dataset. In the case of deep learning models (LSTM, GRU, and SimpleRNN), the grid search was done with the number of hidden units (units list = [32, 64]) and dropout rates (dropout list = [0.1, 0.2]). Validation loss was used to stop early, and the model that best fit on the validation sequences was retained. The look back in time was set at 60-time sequences to ensure that the length of input sequences was similar across different models. In the case of the statistical models, SARIMA parameters (p, d, q) were searched manually between 0 and 2 with a fixed seasonal order, which is one seasonality per week. The scale 0–2 was chosen because previous research and initial experiments have shown that this scale provides a balance between complexity and computational efficiency of models. All combinations of standard additive and multiplicative trend and seasonality were tested in ETS models, and the most efficient model was selected based on the lowest RMSE on the test data. Lastly, the alignment of all model predictions was done on the basis of length and measured on the basis of RMSE, MAE, MAPE, ACF1 of residues, and MASE. This hyperparameter tuning and scaling procedure was systematic, so that there was robust performance, reproducible performance, and comparable performance of models.

## Results

The augmented Dickey‒Fuller (ADF) test was used to determine the stationarity of the time series, and the ADF statistic was −4.69, with a p-value of 8.65e-05. Since the p-value is much lower than the 0.05 significance level, the null hypothesis of non-stationarity can be rejected, and it is established that the time series may be stationary. To develop a model, the data were split into a training set (1333 observations, 69.97%) from March 8, 2020, to January 25, 2024, and a test set (572 observations, 30.03%) from January 26, 2024, to May 24, 2025. The training set was used to fit all models, while the testing set was held out for out-of-sample performance evaluation. A weighted ensemble model was constructed after benchmarking on the test data using the best-performing time series, machine learning, and deep learning models. This model was then applied to predict COVID-19 cases from June 1, 2025, to May 31, 2027.

Table 1 presents the statistics of the daily number of new COVID-19 cases in Bangladesh. The actual observations available include 1905; the mean is 1049.14, and the standard deviation is equal to 2320.55, indicating that the reported daily new cases have considerable variation. The lowest observation is 0, whereas the highest number is 15,810, because there are sometimes drastic changes in the number of cases. It has a very skewed distribution of case counts with the 25th percentile, median (or 50th percentile), and 75th percentile at 8, 49, and 1116, respectively. The skew value of 3.64 further affirms this as the skew is in the right direction, meaning that there are right tails in the distribution, and the kurtosis value of 14.94 implies that it has a heavy tail distribution (typical when there are quick cases increases).

**Table 1:** Summary of daily new COVID-19 cases in Bangladesh (from March 2020 to May 2025)

Daily New Cases	Count	Mean	Std	Min	25%	50%	75%	Max	Skewness	Kurtosis
Bangladesh	1905	1049.14	2320.55	0	8	49	1116	15810	3.64	14.94

**Fig. 2:**
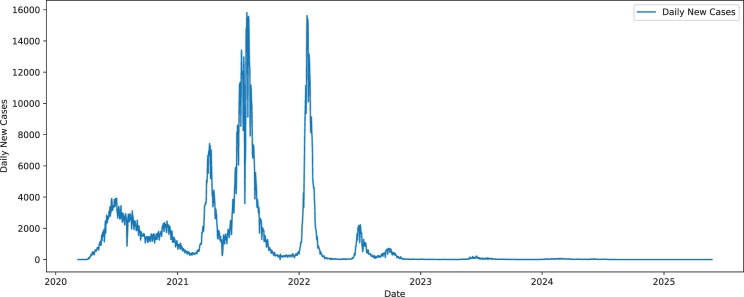
Trends of daily new COVID-19 cases in Bangladesh from March 2020 to May 2025

The time series of daily new COVID-19 cases in Bangladesh between March 2020 and May 2025 is shown in Fig. 2. The figures depict a sequence of sharp spikes related to the primary strategic waves of the pandemic, especially prominent in 2021 and 2022. These spikes indicate sharp changes in daily cases, which are probably caused by mutations of the virus and other social reasons, such as changes in policy, health policy, and vaccination implementation. After the peaks, we can observe an inevitable decline in the number of daily new cases, which means that it is possible for the number of new cases to stabilize as the country progresses through the pandemic. At the end of 2025, with the curve flattening around two and a half, the pandemic situation is described as controlled.

**Fig. 3:**
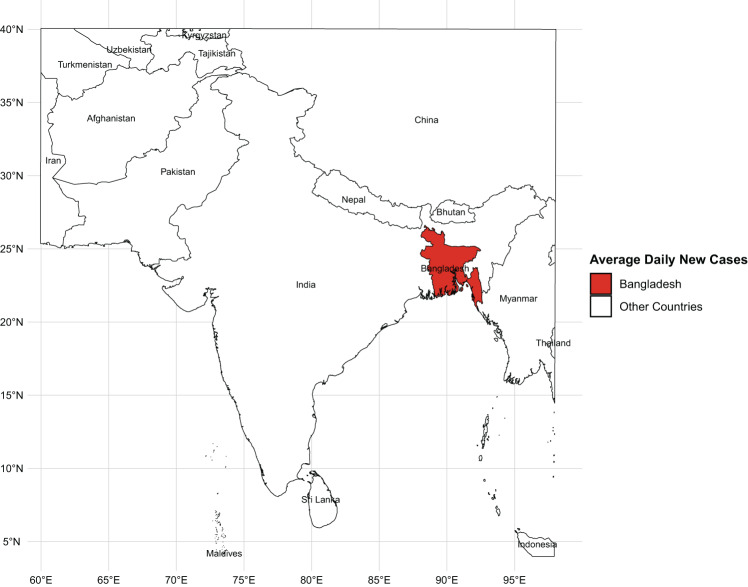
Average daily number of new COVID-19 cases in Bangladesh from March 2020 to May 2025

**Fig. 4:**
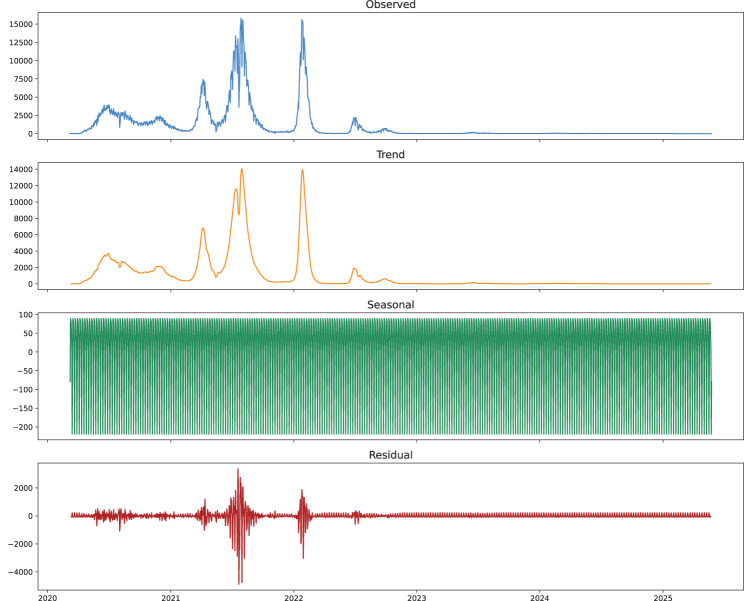
Seasonal decomposition of daily COVID-19 cases in Bangladesh (2020–2025)

The average number of new cases of COVID-19 per day in Bangladesh is presented in Fig. 3, and the country is outlined in red on the map of South Asia. Figure 4 shows the decomposition of the daily COVID-19 cases recorded in Bangladesh from 2020 to 2025, where the given observation data are decomposed into trend, seasonal, and residual parts. The observed series shows that prominent peaks in 2021 and 2022 represent major pandemic waves. The trend component indicates a gradual increase in cases in the early phases of the pandemic, followed by spikes and a certain dip, showing stabilization as the pandemic continued. The seasonal component reflects the consistency in the reverses and implies that case rates are affected by periodic routines or other events. The residual component shows irregular changes not explained by the trend or seasonal changes, concentrating on unusual spikes or differences.

**Fig. 5:**
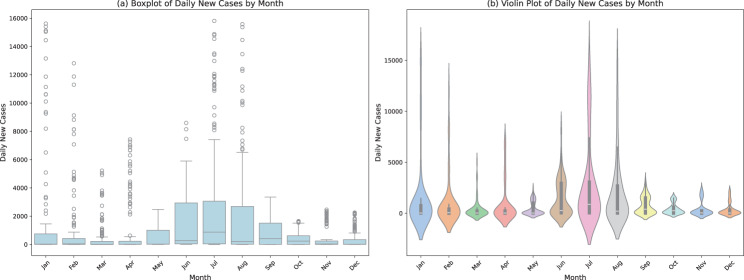
(**a**) Boxplot and (**b**) violin plot of the monthly distribution of daily new COVID-19 cases in Bangladesh

Figure 5 displays the distribution of daily new cases of COVID-19 by month in two forms: a boxplot and a violin plot. The boxplot shows that the variability of the cases is significant, especially in June and July, with the extreme values being highest in July, which amounted to 16,810 instances daily, indicating a remarkable increase. The violin plot shows that there is a sharp peak in July, where the median value is close to 1,200 daily new cases, which points to more cases in that particular month. Its extent is broader in July, which indicates an increase in the number of cases and prolonged duration of high transmission. Such seasonal changes in the middle of the year highlight the need to develop specific public health responses in the months when they are the most predominant.

The results of various forecasting models for predicting new COVID-19 cases per day are presented in Table 2. The table has primary evaluation measures, which are the root mean square error (RMSE), mean absolute error (MAE), mean absolute percentage error (MAPE), autocorrelation function at lag 1 (ACF1), and mean absolute scaled error (MASE). Based on RMSE, the findings indicate that machine learning and deep learning models are much stronger than the implemented traditional statistical approaches. XGBoost has the lowest RMSE value of 5.16, which means that it is the most accurate in terms of minimizing error, as the values of RNN, GRU, and LSTM are very close to RNN (5.37), GRU (5.63), and LSTM (5.73). Conversely, classical models such as SARIMA and ETS exhibit much higher values of RMSE of 14.56 and 17.18, respectively, which indicate poor predictive levels. Other performance indicators can further enlighten: RNN and GRU have the smallest MAE (2.71 and 2.75) and MASE (0.01), and GRU has the smallest MAPE (36.27), which is a sign of good overall performance. SARIMA has a low MAPE (216.87) with a very small MASE (0.04), though it has a high RMSE, indicating that it is not as reliable when measured in percentages. Machine learning models, in particular RNN (−0.14), have lower ACF1, implying less autocorrelation of residues. The combination model, a weighted average (SARIMA, XGBoost, RNN; weights 0.1531, 0.4319, 0.4150), is a better model than SARIMA (14.56) and ETS (17.18), but not its strongest individual parts, including XGBoost (5.16) and RNN (5.37). The combination model indicates also ACF1 = 0.251, MASE = 0.017, MAE = 3.49 and MAPE = 111.02. It offers a steady and consistent performance, leveraging the complementary attributes of its building blocks: SARIMA structure, XGBoost prediction accuracy, and RNN ability to discover patterns over time, although it is not the best-performing model in all its aspects.

**Table 2:** Out-of-sample performance metrics (test data) of the forecasting models for COVID-19 daily new cases

Model	RMSE	MAE	MAPE	ACF1	MASE
SARIMA	14.56	9.02	216.87	0.89	0.04
ETS	17.18	10.41	138.56	0.89	0.05
XGBoost	5.16	3.45	134.52	0.20	0.02
LSTM	5.73	2.96	46.82	0.23	0.01
RNN	5.37	2.71	41.11	−0.14	0.01
GRU	5.63	2.75	36.27	0.02	0.01
Combination	5.87	3.49	111.02	0.251	0.017

Table 2 also indicates high MAPE values (SARIMA: 216.87, ETS: 138.56, XGBoost: 134.52, Combination: 111.02), which is a consequence of 2.94% zero values in the daily COVID-19 cases. According to SARIMA diagnostics (Ljung-Box, *p* = 0.0000; ACF1 = 0.888), there is misspecification; ETS residuals are autocorrelated occurrences (*p* = 0.0000). SARIMA (p,d,q in [0,2], seasonal order (1,1,1,7)) and ETS (all additive and multiplicative trend-seasonality combinations, period = 7) diagnostics (Ljung-Box test, *p* = 0.0000 both SARIMA and ETS, lag = 1) indicate that there is high residual autocorrelation, thus there are non-linear problems in COVID-19 patterns.

**Fig. 6:**
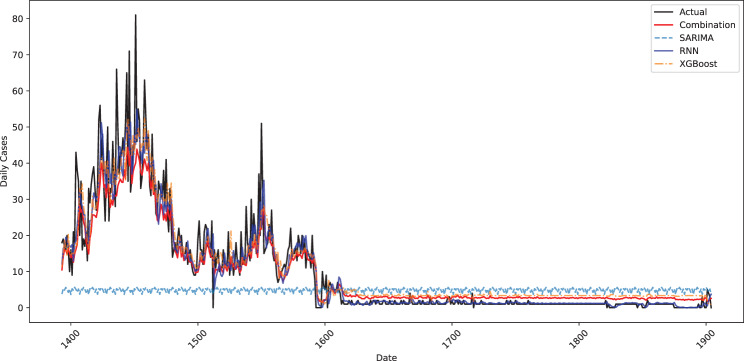
Forecasted plot of daily new COVID-19 cases for test data

Figure 6 compares the actual data on new daily cases of COVID-19 in Bangladesh with the data forecasted by different models. The black line represents the actual data, and the colored lines reflect separate predictions of the model. The SARIMA forecast (sky blue dashed line) shows a smooth, even, slightly poorly fitting estimate, particularly in the case of the peaks. The forecast generated in XGBoost (green dashed line) better captures the changes, but it is less likely to show the peak in the actual number of cases. The RNN forecast (blue line) follows the actual data well at high times and deviates when the situation is at its worst. The combined forecast (red line), a combination of the forecasts of several models, is the most balanced one that reflects both the high and low modes of the data and becomes a better forecast than the separate models do.

## Discussion

**Fig. 7:**
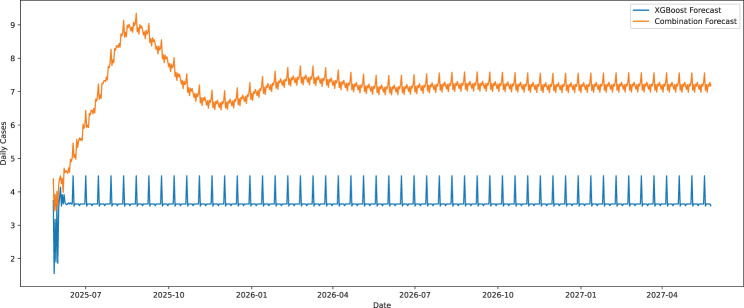
Combined forecast of daily number of new COVID-19 cases in Bangladesh from June 2025 to May 2027 using XGBoost and combination Model

Figure 7 presents the forecast of daily new COVID-19 cases in Bangladesh from June 2025 to May 2027 using the XGBoost model and a combination model (a weighted average of SARIMA, XGBoost, and RNN). XGBoost model ultimately achieves a lower RMSE value on the training set than does the combination model, which is closer to the historical data. However, the weighted ensemble method provided a more realistic daily prediction of total future cases. The XGBoost prediction has a relatively consistent pattern with periodic changes, whereas the combination model reflects a more dynamic pattern, which consists of a seasonal change and long-term trends. Figure 7 also shows that, even though XGBoost is the most effective single model in terms of RMSE, the combination model is more adaptive and robust to predicting the future at the long-term, and thus, it is more appropriate in capturing complex trends of epidemics over time. In practice, a low value of the RMSE for historical observations does not imply that the model will perform ideally in the future, in cases where the model overfits or is sensitive to spurious patterns. With the combination of individual modeling techniques, the combined model likely mitigated the weaknesses of its components, thereby incorporating a broader set of dynamics and classifying them more effectively. This aligns with findings from [36], where an ensemble model using early predictors significantly outperformed individual models in forecasting COVID-19 healthcare demand in France. We also estimated the projections of the June 2025- May 2027 period using our model assessments. These future projections are to provide information to be used as early warning systems, planning of public health, and policy recommendations. The main point is to keep in mind that such predictions are model-based and have uncertainties in data and modeling assumptions, although such may offer a quantitative basis for prediction of potential trends. Thus, one of the main factors to consider in the decisions or treatments is the inclusion of other epidemiological, socioeconomic, and healthcare factors in addition to the model projections.

Hybrid models that combine neural networks with statistical methods, such as AR and LSTM, have improved prediction accuracy by leveraging both linear and nonlinear patterns in a cascaded structure [37]. Similarly, in other papers, deep learning RNN models (LSTM/GRU principal) tend to perform better than classical models of the ARIMA family in COVID-19 time series [38]. An ensemble method incorporating GRU predictions through a weight-averaging mechanism was better for COVID-19 prediction within an Indian setting [39]. These facts confirm our earlier assertions that the key point to remember is that, although the RMSE of the hybrid model is slightly greater than that of the individual models in our analysis, this may be due to the hybrid’s ability to make better generalizations on new data. The pandemic’s peak had greatly diminished worldwide by 2023–2024. The total number of infections exceeds 770 million, and world figures indicate that ~6.9 million have either died or died by August 2023 [40]. Notably, new severe cases and mortality have decreased dramatically in most areas due to increasing vaccination and population immunity, as well as pre-existing immunity. Officially, the World Health Organization claimed that COVID-19 was no longer a Public Health Emergency of International Concern on May 5, 2023, meaning that the global emergency status was finally concluded [40]. Clear improvements in epidemiological trends drove this decision: according to the advisory committee of the WHO, a decrease in mortality due to COVID-19 has been observed over a long period, hospitalization and intensive care admissions are also decreasing, and the population immunity level has reached high rates [41]. These findings indicate that COVID-19 has become a stable, manageable disease and is not becoming an increasing threat to a developing pandemic. At the national level, in Bangladesh, the nature of COVID-19 has followed a downward trend worldwide. Since the onset of the pandemic, Bangladesh has surpassed the 2 million marks of confirmed cases, with approximately 29,000 deaths in total [42]. Nevertheless, towards the end of 2022 and 2023, there was a sharp decrease in the rates of infections and severe consequences in the country. Following several waves (in particular, the delta wave in the middle of 2021 and the Omicron wave in early 2022), the country returned to the phase of low transmission.

Bangladesh’s COVID-19 vaccination campaign, launched in early 2021, achieved one of the highest coverage rates among developing nations, with over 90% of the population receiving full vaccination by mid-2023 [43]. This success resulted from proactive government action, international vaccine procurement, and efficient public health delivery built on the country’s existing immunization infrastructure. Early prioritization of high-risk groups, widespread awareness campaigns, and community engagement help foster vaccine confidence and counter misinformation [44].

COVID-19 is unlikely to resume its global pandemic status on the basis of present statistics and tendencies, developing models, and scientists’ conclusions. Instead, it remains endemic and has some local or seasonal variants, as with other respiratory viruses. We are projecting that case objectives have been reached, with that comprising a functioning, stable, or dropping everyday situation in Bangladesh and not displaying any prospect of an exponential rebound as per the greater scientific gathering that mass immunity has effectively put an end to the period of large-scale worldwide waves [40, 41]. By 2023, it had been estimated that 90–95% of the population would become immune due to vaccination or prior infection, with the potential of the virus declining significantly. Omicron and other sublineages. Another reason is the general lack of virulence of circulating SARS-CoV-2 variants, including the Omicron group, particularly in vaccinated individuals [41]. According to scholars, COVID-19 will stabilize at manageable levels due to fading immunity and seasonal effects, among other factors, without overloading health systems [45].

Our forecasts suggest that projected COVID-19 cases in Bangladesh may remain relatively stable under current trends. However, these results should not be interpreted as a recommendation for specific public health measures, as actual decisions depend on multiple factors including healthcare capacity, vaccination coverage, and emerging variants. Early lockdowns were essential for preventing transmission and providing vaccines, but were associated with significant socioeconomic effects, and their overall cost-effectiveness has become debatable [42]. In the wake of COVID-19, which has become significantly mitigated by widespread immunity, the state of the global health response strategy has shifted away from a more general approach to a much more specific and sustainable countermeasure. The fact that our model does not include exponential growth-predicted solutions indicates that mass lockdowns cannot benefit much. The existing measures are also justified, focusing on vaccination, booster campaigns among vulnerable people, variant monitoring, and the preparedness of healthcare systems. A high level of immunity dramatically reduces the marginal returns of stringent interventions, and excessive aggression can contribute to undue interference.

Our study has several possible limitations, including that other external factors can also stall the model, including the behavior shift of the virus, the appearance of new variants, or developments in public health policy, which may also influence the pandemic course. Despite the fact that the Augmented Dickey-Fuller (ADF) test showed the series to be stationary, visual examination of the series showed complex non-linear alterations, sharp changes, and the long-term correlations. This implies that though the series can be statistically stationary the irregular and non-linear manner in which it behaves can be difficult to explain using summative linear models. As a result, time series models like SARIMA and ETS failed to work, both of them assume stationarity and linearity. Moreover, the sample size (1,905 observations) was relatively small, so the deep learning models such as LSTM, RNN, and GRU could not adequately represent the complex temporal dependencies of the sample, despite the fact that they require larger datasets. Under these circumstances machine learning models especially the XGBoost showed improved performance because it was flexible to model non-linear relationships. More advanced models such as Support Vector Regression (SVR), Random Forest, and CatBoost were not investigated due to the unwarranted complexity, but they can be the potential sources of future research on how to make the forecast more accurate. Lastly, the popular MAPE measure is also susceptible to zero and small values (2.94 percent zero in this dataset) which will overreact to error values, especially of SARIMA and ETS predictions. It is thus suggested that in future work, weighted MAPE (WMAPE) should be used to offer a more accurate evaluation of the performance of the model.

## Conclusion

In this work, it can be noted that a great deal of success was achieved in predicting the number of new COVID-19 cases in Bangladesh per day during 8 March 2020 to 25 May 2025, having 1905 cases with 2.94% of zero-case days. Seasonal Autoregressive Integrated Moving Average (SARIMA), XGBoost, and Recurrent Neural Network (RNN) as single-model forecasts and a Weighted Ensemble forecasting model with the combination of those were. The ensemble approach performs better than single models, and it has attributes of RMSE = 5.87, MAE = 3.49, and MAPE = 111.02, even when non-linear and complex patterns are present, and over-model specification is a common occurrence in the traditional models. To our knowledge, a weighted ensemble framework applied in this work is the first study that made univariate predictions of COVID-19 cases in Bangladesh and is more accurate and robust. The results have a practical implication on policymakers, as they can use them to maximize resources, micro-manage interventions, and to prevent unnecessary lockdowns as COVID-19 transitions to endemicity. Moreover, we predict the COVID-19 cases between June 2025 and May 2027 to have an early warning mechanism to enable people to take the relevant precautions, and the government and policymakers to adopt appropriate measures in time. This also aids in the aspect of planning for economic backup strategies and health preparedness. To improve these forecasts, future studies may implement more interpreters of the model, e.g., Shapley values, or other evaluation measurements, e.g., weighted mean absolute percentage error, to correct data sparsity and increase the confidence of making decisions in the field of public health.

## Data Availability

All data supporting the findings of this study were obtained from Our World in Data (https://ourworldindata.org/) and are openly available. The processed dataset used in this study is available from the corresponding author (A.K.D.) upon reasonable request.
